# Antioxidant, Antibacterial, and Cytoprotective Activity of Agathi Leaf Protein

**DOI:** 10.1155/2014/989543

**Published:** 2014-01-28

**Authors:** A. S. Zarena, Shubha Gopal, R. Vineeth

**Affiliations:** Department of Studies in Microbiology, University of Mysore, Mysore 570006, India

## Abstract

In the present study a protein termed agathi leaf protein (ALP) from *Sesbania grandiflora Linn*. (agathi) leaves was isolated after successive precipitation with 65% ammonium sulphate followed by purification on Sephadex G 75. The column chromatography of the crude protein resulted in four peaks of which Peak I (P I) showed maximum inhibition activity against hydroxyl radical. SDS-PAGE analysis of P I indicated that the molecular weight of the protein is **≈**29 kDa. The purity of the protein was 98.4% as determined by RP-HPLC and showed a single peak with a retention time of 19.9 min. ALP was able to reduce oxidative damage by scavenging lipid peroxidation against erythrocyte ghost (85.50 ± 6.25%), linolenic acid (87.67 ± 3.14%) at 4.33 **μ**M, ABTS anion (88 ± 3.22%), and DNA damage (83 ± 4.20%) at 3.44 **μ**M in a dose-dependent manner. The purified protein offered significant protection to lymphocyte (72% at 30 min) induced damage by t-BOOH. In addition, ALP showed strong antibacterial activity against *Pseudomonas aeruginosa* (20 ± 3.64 mm) and *Staphylococcus aureus* (19 ± 1.53 mm) at 200 **μ**g/mL. The safety assessment showed that ALP does not induce cytotoxicity towards human lymphocyte at the tested concentration of 0.8 mg/mL.

## 1. Introduction

Plants contain a huge range of active compounds with the most abundant being polyphenols, carotenoids, vitamin, and metals like zinc and selenium which form an integral part of antioxidant systems and reduce cellular damages. In addition fruits and vegetables are often low in fat and therefore dietary sources have been recognized as safe and effective antioxidants. In recent years considerable effort has been directed towards the search for safe antioxidants from natural sources in context to their efficiency and nontoxicity.

*Sesbania grandiflora* also known as agathi belongs to the family Fabaceae. It is a fast growing tree and is widely distributed in India, Indonesia, Myanmar, Philippines, and Thailand. The tree grows 5–15 m tall and the leaves and flowers of this tree are eaten as nutrition source. The leaves are bitter in taste and are rich in vitamin C, calcium, sterols, saponin, quercetin, myricetin, and kaempferol [1]. The leaves of the agathi are well known for their antiurolithiatic activity against calcium oxalate-type stones [2]. In a recent study, China et al. [3] have reported antimicrobial property of polyphenolic extract of *S. grandiflora* on pathogenic bacteria and growth promoting effect on *Lactobacillus acidophilus*. Boonmee et al. [4] have isolated two unique proteins (SGF60 and SGF90) from the flower extract of agathi showing *α*-glucosidase inhibiting property. Laladhas et al. [5] have isolated a protein fraction (*Sesbania* fraction 2) from the flower of *S. grandiflora *which possesses anticancer efficacy. We herein report the details of our study leading to the isolation and purification of a novel *≈*29 kDa protein from *S. grandiflora* leaves. The newly isolated protein hereafter called agathi leaf protein (ALP) was tested *in vitro* for antioxidant, cytoprotective, and antibacterial activity.

## 2. Experimental

### 2.1. Materials

BHA (butylated hydroxyanisole), N,N,N′,N′-tetramethylethylenediamine (TEMED) bisacrylamide, 2,2-azinobis(3-ethylbenzothiazoline-6-sulfonic acid) diammonium salt (ABTS), and 5,5′-dithiobis-(2-nitrobenzoic acid) (DTNB) were from Sigma Chemicals (St. Louis, MO, USA). t-BOOH (tertiary butylated hydroperoxide) and Sephadex G 75 were purchased from Pharmacia, Sweden. Ferric chloride, hydrogen peroxide (H_2_O_2_), ferrous sulphate, ascorbic acid, potassium persulfate, ethylenediaminetetraacetic acid (EDTA), thiobarbituric acid (TBA), polyvinyl pyrrolidone, and 2-deoxyribose were purchased from Merck (Mumbai, India). Calf thymus DNA was from Himedia Private Ltd. (Mumbai, India). All other reagents used were of analytical grade.

The plant sample of *Sesbania grandiflora* (Family: Fabaceae) was collected from Mysore (Karnataka), India. The fresh uninfected leaves were washed in autoclaved water to remove extraneous material, air-dried in an open space at aseptic condition for about 10–15 days, ground to fine powder, and stored at 4°C overnight until further use.

### 2.2. Bacterial Strains

Bacterial strains used in the study were *Staphylococcus aureus* ATCC 12600 (Gram-positive bacteria), *Salmonella typhimurium* ATCC 13311,* Escherichia coli* ATCC *11775*, *Vibrio parahaemolyticus* ATCC 17802, *Klebsiella pneumoniae* ATCC 10031, and *Pseudomonas aeruginosa* ATCC 10145 (Gram-negative bacteria).

### 2.3. Preparation of the Agathi Leaf Protein Extract

Five grams of agathi leaf powder was added to 50 mL of hot double-distilled water; to this 100 mg of polyvinyl pyrrolidone was added to remove polyphenols. The resultant solution was homogenized and incubated overnight at 4°C. The supernatant was centrifuged at 10,000 rpm for 15 min at 4°C (refrigerated centrifugation) and was filtered through Whatman number 1 filter paper. The above crude extract was precipitated with 0–80% ammonium sulphate and dialyzed against double-distilled water for 3 days with four changes. The precipitates were pooled by centrifugation at 10,000 rpm for 15 min and resuspended in double-distilled water and dialysed to desalt (NH_4_)_2_SO_4_. The solution was concentrated and fractionated on Sephadex G 75 using Tris-HCl buffer (25 mM, pH 7.4) as eluent (1 g crude protein, *V*_*o*_ 35.4 mL, *V*_*t*_ 114 mL, and flow rate 1.5 mL/5 min). Each fraction was monitored at 280 nm and the protein content was estimated by Bradford's method [6]. The peak fractions (Peak I) which had maximum antioxidant activity were pooled, lyophilized, and rechromatographed on Sephadex G 75 column for further analysis.

### 2.4. Proximate Analysis

#### 2.4.1. Estimation of Protein Content

The total protein content of the crude extract was determined by Bradford's [6] method. Various concentrations of bovine albumin (0–100 *μ*g/mL) or agathi leaves extract at the concentration ranging from 0 to 20 *μ*L were added to series of tubes and the volume was made up to 100 *μ*L with 0.15 M NaCl. 1 mL Bradford's reagent was added to all the tubes and mixed well. The absorbance was measured at 595 nm. The concentration of the protein in the samples was determined from the calibration curve.

#### 2.4.2. Estimation of Total Sugar

The total sugar was estimated by the phenol-sulphuric acid method [7]. Different aliquots of the extract (0–25 *μ*L) were made up to 1 mL with distilled water. To this 1 mL of 5% phenol and 5 mL of concentrated sulphuric acid were added. Orange color developed was read at 520 nm immediately. The sugar concentration of the extract was calculated according to the standard glucose calibration curve.

#### 2.4.3. Determination of SH Groups

Sulphydryl group was estimated by Ellman's method [8]. 5-6 mg of agathi leaf protein extract was taken in 2 mL of phosphate buffer (0.1 M, pH 8.0), to this 0.4% 5,5′-dithiobis-(2-nitrobenzoic acid) (DTNB) of aqueous solution was added and mixed well. Absorbance was measured at 412 nm after 1 min. The concentration of the sample was determined using the formula

(1) $$\begin{array}{c} C_{o}=\frac{A}{ED}, \end{array}$$

where *C*_*o*_ is the concentration of the sample, *A* is the absorbance at 412 nm, *E* is the molar extinction coefficient of 13,600 M^−1 ^cm^−1^, and *D* is the dilution factor.

#### 2.4.4. Determination of Total Phenol Content

The total phenolic content was determined according to the method of Folin-Ciocalteu reaction [9] with minor modifications, using gallic acid as standard. An aliquot of the samples (10–40 *μ*L) was mixed with 50% Folin-Ciocalteu reagent; the volume was made up to 1 mL with methanol: water mixture (50 : 50 v/v). The mixture was then allowed to stand for 10 min followed by the addition of 20% Na_2_CO_3_. After 10 min incubation at ambient temperature, absorbance was measured at 725 nm. Results were expressed as milligrams of gallic acid equivalents (GAE) per gram.

#### 2.4.5. Estimation of Total Chlorophyll

The total chlorophyll content was determined according to the method of Sadasivam and Manickam [10] with minor modifications. 1 g of agathi leaves was ground in a clean mortar and pestle with 15–20 mL of 80% acetone and centrifuged at 5000 rpm for 10 min and supernatant was collected. This procedure was repeated several times till a clear supernatant was obtained and the volume was made up to 100 mL with 80% acetone. The absorbance of the solution was read at 645 nm and 663 nm against solvent (80% acetone) blank. The amount of total chlorophyll present in the extract was calculated using the following equation:

(2) mg  chlorophyll/g  extract=20.2  (A645)+8.02  (A663) ×V100×V,

where *A* is the absorbance at specific wavelengths, *V* is the final volume of chlorophyll extract in 80% acetone, and *W* is the dry weight of extract.

#### 2.4.6. Test for Protein

Agathi leaf protein was spotted on chromatography paper, sprayed with 0.2% solution of ninhydrin (indane-1,2,-3-trione hydrate), and dried. The appearance of purple/violet color spot indicated the presence of protein.

### 2.5. Determination of Molecular Weight and Purity Check

Polyacrylamide slab gel (12% acrylamide in separating gel and with 4% in stacking gel) was prepared and electrophoresis was performed as described by Laemmli [11]. The samples were mixed with sample buffer containing glycerol, sodium dodecyl sulfate (SDS) in Tris buffer (pH 8.3), and bromophenol blue as tracking dye. Prior to electrophoresis, the samples were incubated at 95°C for 5 min. Gel was run at 50 V for stacking and 100 V for separating gel. The bands were stained with coomassie brilliant blue-250 and destained in methanol/acetic acid/water (5/1/5; v/v/v).

A reversed-phase high-performance liquid chromatographic was performed for evaluation of purity of the isolated protein. Separation of the peak was accomplished on a Phenomenex C-18 column 250 mm × 4.60 mm i.d.; particle size, 5 mm at ambient temperature using 0.1% formic acid in acetonitrile : methanol (75 : 25) as mobile phase in an isocratic elution mode. A photodiode array detector set at 280 nm was used for detection.

### 2.6. Hydroxyl Radical Scavenging Activity

Deoxyribose assay was used to determine the hydroxyl radical scavenging activity according to the method of Chung et al. [12] with some modification. The reaction mixture contained FeCl_3_ and ascorbate (100 *μ*M), H_2_O_2_ (1 mM), EDTA (100 *μ*M), 2-deoxy-D-ribose (2.8 mM), and 1 mL of 0.1 mM potassium phosphate buffer (pH 7.4) mixed in various concentrations of ALP extract (50−400 *μ*g/mL). The reaction mixture was incubated for 1 hour at 37°C. The reaction was terminated by adding 1 mL each of TCA (2.8%) and TBA (0.5%); this mixture was placed in boiling water bath for 15 min. After cooling, the reaction mixture was centrifuged for 5 min at 5000 rpm. The control was without any test compound and the readings were taken at 535 nm. The percentage hydroxyl radical scavenging activity was determined by comparing with control. Decreased absorbance of the reaction mixture indicated decreased oxidation. Consider the following:

(3) %    Inhibition=AbsorbanceControl−AbosorbanceTestAbsorbanceControl ×100.

### 2.7. Determination of Antioxidant Activity Using Erythrocyte Ghost and Linolenic Acid Micelles

Erythrocyte membranes (ghosts) preparation was carried out according to the method of Dodge et al. [13] with modifications. Fresh heparinised human blood samples were drawn with anticoagulant (acid citrate dextrose) and centrifuged at 2500 rpm for 10 min at 4°C; the supernatant was discarded and the pellet was washed 3–5 times with isotonic phosphate buffer (PBS 5 mM, pH 7.4, and 150 mM NaCl). The RBC cell pellet was suspended in hypotonic phosphate buffer (PBS 5 mM, pH 7.4 at 4°C) for hemolysis to take place. Contents were centrifuged at 12,000 rpm at 4°C for 20 min. Erythrocytes were separated from plasma and buffy coat and buffy cat was washed with fresh hypotonic phosphate buffer centrifuged at 1500 rpm for 10 min to remove unlysed RBC cells. Finally, the membranes were resuspended in isotonic 5 mM phosphate buffer, pH 7.4, to yield a dispersed pale yellowish pink “ghost.” The protein content of ghost was estimated by Bradford's method [6]. Ghost suspension (200 *μ*g) and linolenic acid (1.8 *μ*mole) were subjected to peroxidation by ferrous sulphate and ascorbic acid (10 : 100 *μ*mole) in a final volume of 0.5 mL Tris buffered saline (TBS 100 mM, pH 7.4, and 0.15 M NaCl) with increasing concentration of ALP extracts (0.86–4.33 *μ*M); the contents were incubated at 37°C for 1 h; to this 1% TBA was added; and the contents were kept in a boiling water bath for 15 min and then cooled, centrifuged to remove precipitate if any. The color developed was measured at 535 nm (see (3))

(4) %    Inhibition=AbsorbanceControl−AbosorbanceTestAbsorbanceControl ×100.

### 2.8. DNA Sugar Damage by Spectrophotometric Method

Oxidative DNA sugar damage was induced with Fenton's reactants and was determined according to the method of Cao et al. [14]. The reaction mixture in a total volume of 1 mL containing 1 mg calf thymus DNA was treated with Fe^3+^ (10 mM), EDTA (10 mM), and H_2_O_2_ (2 mM) without or with various concentrations of the extract (0.68–3.44 *μ*M) in potassium phosphate buffer (20 mM, pH 7.4). Ascorbic acid (10 mM) was added to the reaction mixture and was incubated at 37°C for 1 h in water bath with shaker. To 1 mL of the above mixture 1 mL, of TCA and 1 mL of 1% TBA were added and boiled for 20 min. The contents were cooled and the pink color absorbance was read at 523 nm (see (3))

(5) %    Inhibition=AbsorbanceControl−AbosorbanceTestAbsorbanceControl ×100.

### 2.9. Scavenging of ABTS^●+^ Radical

ABTS radical cation decoloration assay was performed according to Re et al. [15] with some modifications. ABTS stock solution was prepared by reacting 7 mM ABTS with an oxidant 2.45 mM potassium persulfate in dark, at room temperature, for 12–16 h before use. Prior to the assay, the solution was diluted in water and equilibrated at room temperature to give an absorbance of 0.70 ± 0.02 at 734 nm. Different volumes of the sample (0.68–3.44 *μ*M) were mixed with 3 mL of ABTS^●+^ solution and absorbance at 734 nm was measured. BHT was used as positive control. The scavenging activity was calculated using the following equation:

(6) %    Inhibition=AbsorbanceControl−AbosorbanceTestAbsorbanceControl ×100.

### 2.10. Lymphocyte Isolation and Protection Study

Human peripheral lymphocytes were isolated according to the method of Smitha et al. [16]. To 10 mL of venous blood, four volumes of hemolysing buffer (150 mM NH_4_Cl in 10 mM tris buffer, pH 7.4) were added and mixed well; the contents were incubated at 4°C for 30 min and centrifuged at 1200 rpm for 12 min, the supernatant was discarded, and pellet was washed thrice with 10 mL of 250 mM m-inositiol in 10 mM phosphate buffer, pH 7.4, and resuspended in the same solution. The cell viability was determined by trypan dye blue exclusion method. Percentage viability was calculated as

(7) $$\begin{array}{c} \%\;\;\text{Viability}=\frac{\text{Number}\;\;\text{of}\;\;\text{viable}\;\;\text{cells}}{\text{Total}\;\;\text{number}\;\;\text{of}\;\;\text{cells}}\times 100. \end{array}$$

The isolated lymphocyte was subjected to lipid peroxidation by t-BOOH (1 mM) in the presence of ALP (6.8 *μ*M) and BHA (400 *μ*M) in a reaction mixture of 1 mL buffered with Hanks' buffer saline solution (HBSS), pH 7.4, and incubated at 37°C. To 10 *μ*L of lymphocyte sample, 100 *μ*L of tryphan blue (1%) was added and the viable cells (unstained) were counted using Neuber's chamber. (The dead cells being permeable to tryphan blue appear blue against white color of the viable cells.) The survival rate of lymphocyte was determined at time intervals 15, 30, 60, 180, and 300 min of incubation.

### 2.11. Cytotoxicity Study

Cell suspensions were incubated with different concentrations of ALP (0–0.8 mg/mL) for 30 min at 37°C in dark together with untreated control samples. Samples were then centrifuged at 2700 rpm, the lymphocytes were suspended in 0.9% saline and 1% trypan blue, and viable and dead cells were observed.

### 2.12. Antibacterial Activity of ALP

Antibacterial activity of the extract was determined by the disc diffusion method. 100 *μ*L of overnight bacterial culture in Tryptic soy broth, adjusted to 0.4-0.5 Mc Farland turbidity (10^4^ CFU/mL), was used as inoculum. The suspension was homogenously swabbed on Muller-Hinton agar media (MHA) using sterile cotton swab. The extract (100–200 *μ*g/mL) was loaded on sterile disc (6 mm), placed on MHA medium containing the culture, and allowed to diffuse for 15 min. The plates were kept for incubation at 37°C for 24 hrs and the inhibition zones formed around the disc were measured in mm. Gentamicin (10 *μ*g) disc was used as positive control and negative control was 20 mM Tris buffer saline.

### 2.13. Experimental Design and Statistical Analysis

Our present study was classified into two stages. The first stage was to isolate and purify the protein from agathi leaf extract. The second stage was to fix the effective concentration of ALP to carry out antioxidant, antimicrobial, and cytotoxicity study. Statistical analysis was carried out using Statistical Package for Social Science (SPSS, version 20.0). The experimental results were expressed as the mean ± standard deviation (*n* = 3). Group comparisons were performed using one-way ANOVA followed by Tukey's post hoc test. A *P* value of 0.05 was considered statistically significant.

## 3. Results and Discussion

**Figure 1:**
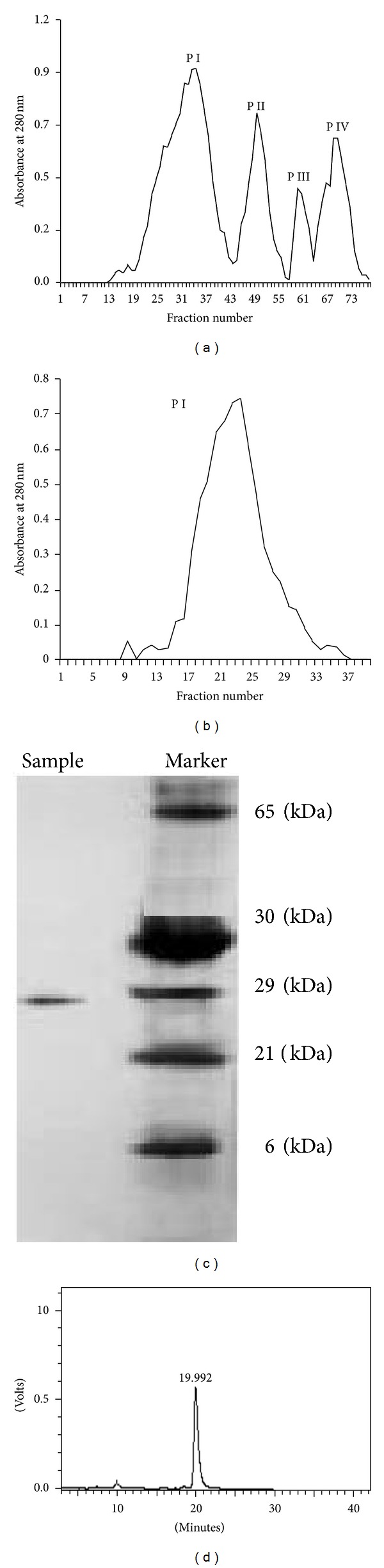
(a) Agathi leaf protein fractionated on Sephadex G-75 column and the fractions monitored at 280 nm. (b) Rechromatography of Peak I on Sephadex G-75 and the fractions monitored at 280 nm. Fractions were obtained at a flow rate of mL/min using Tris-HCl buffer. (c) SDS-PAGE analysis of Peak I yielding a single band of *≈*29 kDa. (d) RP-HPLC of Peak I on Phenomenex C-18 column 250 mm × 4.60 mm i.d.; particle size, 5 mm.

In the present study, 65% ammonium sulphate precipitation of the crude agathi leaf protein on their subsequent fractionation through sephadex G 75 column yielded four peaks that were designated as P I, II, III, and IV (Figure 1(a)). P I showed a maximum hydroxyl scavenging activity up to 83% at 100 *μ*g followed by P III 54% at 250 *μ*g, whereas P II and P I showed 38% and 32% activity at 400 *μ*g, respectively. P I fraction that showed maximum antioxidant activity was pooled separately, lyophylised, and rechromatographed through Sephadex G 75, yielding a single peak (Figure 1(b)). Further the homogeneity and the purity of P I were confirmed by SDS-PAGE that resulted in a single band with approximate molecular weight of *≈*29 kDa (Figure 1(c)). The molecular mass of the purified protein was estimated by comparison with the molecular mass of the marker protein. The HPLC chromatogram showed a single peak with a retention time of 19.9 min in isocratic mode and purity was 98.4% (Figure 1(d)). P I was designated as agathi leaf protein (ALP). The proximate analysis of the ALP extract proved to be positive for ninhydrin. There was no presence of polyphenols and chlorophyll. The total sugar was found to be 0.188 mmol. The test for sulphydryl group was positive with 6 *μ*mol of sulfhydryl groups/g being detected.

**Figure 2:**
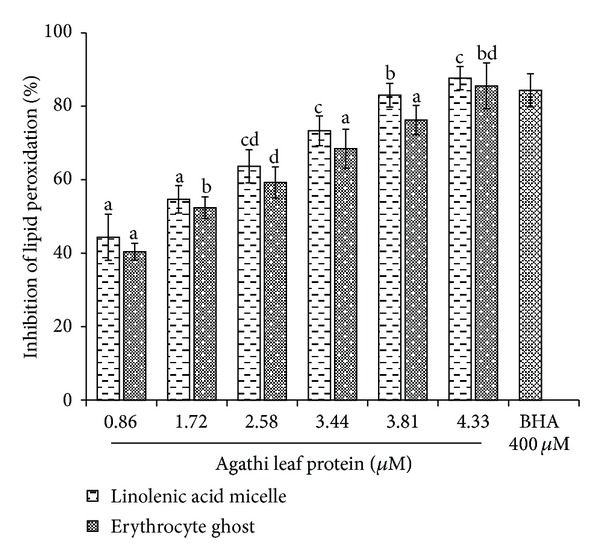
Inhibition of lipid peroxidation in linolenic acid micelle and erythrocyte ghost. Data are expressed as the mean ± standard deviation (*n* = 3). Means with different letters (a–d) are significantly different (*P* < 0.05).

The *in vitro *peroxidation of human erythrocyte ghosts and linolenic acid micelles was used as a model system to study the free radical induced damage of biological membranes and the protective effect of ALP. Membrane lipids being rich in unsaturated fatty acids especially linoleic, linolenic, and arachidonic acids when attacked by free radicals form lipid peroxide. In the present study the antioxidant activity of the ALP was studied in comparison with known antioxidant like BHA. It was observed that the inhibitory effect of ALP in erythrocyte ghost and linolenic acid micelles was found to be 85.50 ± 6.25% and 87.67 ± 3.14% at 4.33 *μ*M dose dependently compared to BHA which showed an activity of 84.33 ± 4.50 at 400 *μ*M (Figure 2). Erythrocyte ghost and linolenic acid micelles are simple suitable model system commonly used in the study of LOP as the protein composition of the former is well known and they lack organelles [17]. On the other hand, linolenic acids are present in food and organisms and their oxidation results in the formation of hydroperoxides.

**Figure 3:**
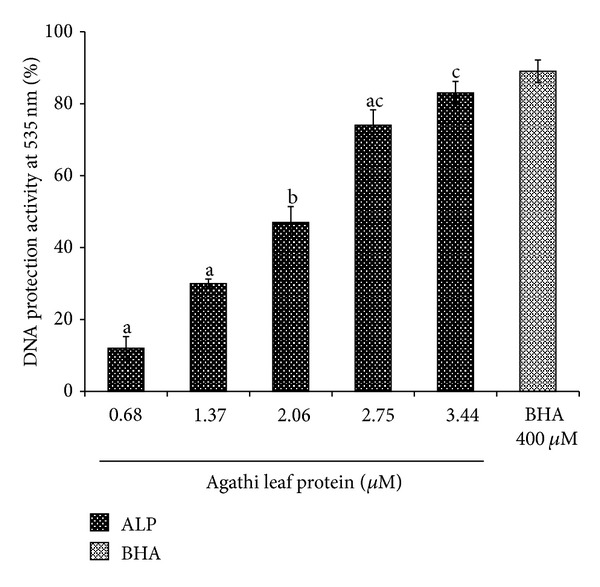
Inhibition of hydroxyl radical-mediated DNA degradation by ALP. Data are expressed as the mean ± standard deviation (*n* = 3). Means with different letters (a–c) are significantly different (*P* < 0.05).

**Figure 4:**
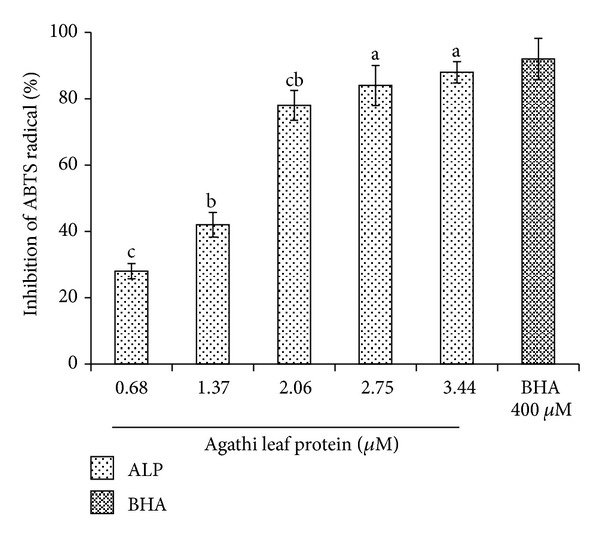
Scavenging effect of ALP on ABTS radical. Mean values ± standard deviations (*n* = 3) with the same letter are not significantly different (*P* < 0.05).

The effect of ALP on hydroxyl radicals generated by Fe^3+^/H_2_O_2_ ions was measured by determining the degree of DNA degradation by test tube assay (Figure 3). While a marginal inhibition was evident at the lower concentration, nearly 83 ± 4.20% inhibition was observed at higher concentration at 3.44 *μ*M. The scavenging effect increased with increasing ALP concentration up to a certain extent (3.44 *μ*M) and then leveled off with further increase. Further ABTS^●+^ method showed an activity of 88 ± 3.22% at 3.44 *μ*M when compared to synthetic antioxidant BHA which showed an activity of 92 ± 4.03% at 400 *μ*M (Figure 4) and leveled off thereafter. It was observed that the inhibition value of ALP increased with increase in concentration.

**Figure 5:**
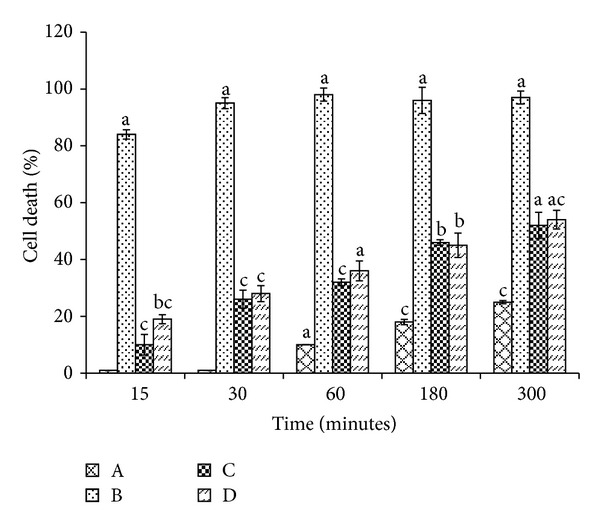
Prevention of t-BOOH induced cell death in lymphocytes by ALP and BHA. A: lymphocytes; B: lymphocytes + t-BOOH (1 mM); C: lymphocytes + t-BOOH (1 mM) + BHA (400 *μ*M); D: lymphocytes + t-BOOH (1 mM) + ALP (6.88 *μ*M). Data are expressed as the mean ± standard deviation (*n* = 3). Means with different letters (a–c) are significantly different (*P* < 0.05).

We also investigated the lipid peroxidation induced cell death by t-BOOH (Figure 5). Treatment with t-BOOH on lymphocyte cells significantly showed cell toxicity. The maximum cell death was induced by t-BOOH at 30 min, while the cells incubated with BHA (400 *μ*M) or ALP (6.88 *μ*M) showed an increase in cell viability. The protection offered by ALP was 72% and BHA was 74% at 30 min. As the time of incubation period increased, the percentage of cell death increased. The differences in antioxidant activity in the above assays could probably be due to the different mechanisms occurring in the assay, varying sensitivity of the assay system, and their concentration-dependent activities.

The protective effect of ALP toward human lymphocytes is shown in Table 1. The cell viability was greater than 95% at the concentrations tested (0–0.8 mg/mL). The high percentage of viable cell clearly indicates that ALP is a nontoxic protein with no cytotoxicity toward human lymphocytes.  Table 2 shows the antimicrobial screening of ALP against Gram-positive and Gram-negative bacteria. ALP extract showed maximum antibacterial activity against *S. aureus, K. pneumonia*,and* P. aeruginosa* with zone of inhibition ranging from 15 to 20 mm at 200 *μ*g/mL and no activity against* S. typhimurium* at the same concentrations. The most susceptible bacterium was *P. aeruginosa* ATCC 10031 (20 ± 3.64 mm diameter). The extract showed lower sensitivity in comparison to the positive control gentamicin. The antioxidant and antimicrobial activity of ALP could be attributed to the presence of cysteine/cystine and the occurrence of disulphide bridge [18] as determined by Ellman's test. In the present study, Ellman's test for “S–S” group proved to be positive indicating the presence of cysteine/cystine residues. SH group acts as free radical scavenger in plants and animals and facilitates the antioxidant activity of glutathione [19].

## 4. Conclusions

The results obtained in the present study demonstrate ALP obtained from water extract of agathi leaf can effectively scavenge various ROS *in vitro* conditions at low dose. ALP showed strong inhibitory activity toward lipid peroxidation on RBC ghost and linolenic acid micelle system. Furthermore, ALP exhibited a strong concentration-dependent inhibition against deoxyribose oxidation and DNA damage. ALP is not only interesting source for antioxidant but also potential source of antimicrobial agent and nontoxic in nature. The present study showed that the investigated proteins are promising ingredients for the development of functional foods with a beneficial impact on human health and an important source for the production of bioactive proteins.

## Figures and Tables

**Table 1:** Cytotoxicity of ALP extract toward human blood lymphocytes.

Concentration (mg/mL)	Viability (%)
0	98.0 ± 3.42^c^
0.2	98.0 ± 1.34^c^
0.4	96.0 ± 1.77^a^
0.6	98.0 ± 1.86^c^
0.8	97.0 ± 0.91^b^

Data are expressed as the mean ± standard deviation (*n* = 3). Means with different letters (a–c) are significantly different (*P* < 0.05).

**Table 2:** Antimicrobial activity of ALP (zone size, mm).

Test bacteria	ALP (100 *μ*g/mL)	ALP (200 *μ*g/mL)	Gentamicin (10 *μ*g)
*Staphylococcus aureus* ATCC 12600	—	19 ± 1.53^a^	26 ± 0.25^a^
*Salmonella typhimurium* ATCC 13311	—	—	15 ± 0.97^c^
*Escherichia coli* ATCC 11775	13 ± 1.31^c^	15 ± 1.24^a^	17 ± 0.48^a^
*Vibrio parahemolyticus* ATCC 17802	—	15 ± 2.20^b^	18 ± 0.49
*Klebsiella pneumoniae* ATCC 10031	11 ± 0.40	18 ± 1.61^b^	22 ± 0.83^d^
*Pseudomonas aeruginosa* ATCC 10145	18 ± 0.86^d^	20 ± 3.64^c^	21 ± 0.62^a^

(—): no inhibition.

Data are expressed as the mean ± standard deviation (*n* = 3). Means with different letters (a–d) within the same column are significantly different (*P* < 0.05).
